# Therapeutic mechanisms of Zi Chong granules against hydroxyurea-induced diminished ovarian reserve based on integrated multi-omics analyses

**DOI:** 10.1186/s13048-025-01846-5

**Published:** 2025-12-16

**Authors:** Wenran Dong, Xinyu Guo, Hua Lu, Zhibin Liu, Lan Xie, Yi Liu, Qian Wan, Ren Chen, Sui Liu

**Affiliations:** 1 Chengdu University of Traditional Chinese Medicine, Chengdu, China; 2 Key Laboratory of Bio-Resource and Eco-Environment of Ministry of Education, College of Life Sciences, Sichuan University, Chengdu, China; 3 Medical Systems Biology Research Center, School of Medicine, Tsinghua University, Beijing, China; 4 Chengdu Neo-Life Hope Medical Testing Lab. Co. Ltd, Chengdu, China; 5 China Resources Sanjiu Medical & Pharmaceutical CO., LTD, Huizhou, China; 6 Traditional Chinese Medicine Hospital of Qionghai, Qionghai, China; 7 National Engineering Research Center for Beijing Biochip Technology, Beijing, China; 8 Beijing University of Chinese Medicine, Beijing, China

**Keywords:** Zi chong granules, Hydroxyurea, Diminished ovarian reserve, Gut microbiome, Metabolomics

## Abstract

**Background:**

Hydroxyurea (HU) is an antitumor drug. However, HU exposure is associated with diminished ovarian reserve (DOR). Zi Chong granules, a Chinese Medicine, can protect against DOR, but little is known regarding its underlying mechanisms of DOR treatment, and thus the target of the present study.

**Methods:**

Seventy-two female Kunming (KM) mice (4–6 weeks old) were randomly divided into three groups: the control group (Con), the hydroxyurea group (HU), and the Zi Chong group (ZC). The Con group received saline, while the HU and ZC groups were administered hydroxyurea (400 mg/kg/d) by gavage for 21 days to induce diminished ovarian reserve (DOR). Subsequently, the Con and HU groups were given saline, while the ZC group was treated with Zi Chong granules (2.72 g/kg/d) for 15 days. Ovaries and uterus of mice were examined histologically by H&E. The levels of anti-Mullerian hormone (AMH), estradiol (E_2_), and progesterone (P) were quantified using ELISA kits. The number and quality of oocytes were assessed, and endometrial receptivity was evaluated by immunohistochemistry. 16 S rDNA gene sequencing was used to analyze the composition and abundance of gut microbiome in feces, and non-targeted metabolomics was performed to detect serum metabolite profiles. Correlation analysis was performed to explore the relationships between different gut microbiota and differential metabolites.

**Results:**

ZC granules increased the number of primordial follicles in the ovaries, reduced excessive follicular atresia, restored low AMH, upregulated estrogen and progesterone secretion, and increased the number of mature oocytes after ovulation promotion. It also increased thickness of uterine endometrium and the number of glands, resulting in increased endometrial microvessel density (MVD), enhanced endometrial blood supply, reduced CD138 expression levels and endometrial inflammation. HU decreased the abundance of *Lactobacillus spp.* in mouse intestines and decreased arachidonic acid metabolism, tryptophan metabolism, spermidine and spermine biosynthesis, steroidogenesis, and nicotinate and nicotinamide metabolism. Correlation analysis revealed that HU exerted its side effects by altering the gut microbiome and bacteria-derived metabolites, while ZC granules could reverse DOR partly depends on regulating gut microbiota and metabolites.

**Conclusions:**

ZC granules may be a potential therapy for alleviating HU-induced DOR.

**Supplementary Information:**

The online version contains supplementary material available at 10.1186/s13048-025-01846-5.

## Background

Hydroxyurea (HU) is an oral chemotherapeutic agent that is widely used to treat various diseases, including sickle cell disease, chronic granulocytic leukemia, and other cancers. However, its clinical use can result in side effects, such as bone marrow suppression and anemia, as well as toxic effects on the human reproductive system, including diminished ovarian reserve (DOR) in women. A multicenter study of patients with sickle cell disease treated with HU and followed for 10 years found that its administration was strongly associated with low anti-Müllerian hormone (AMH) levels, leading to premature decline of ovarian reserve [1]. Another study found a significant decrease in the antral follicle counts (AFC) in the ovaries of women exposed to HU compared with controls [2]. HU also caused reduced ovarian reserve function, as well as causing embryonic cell death and embryonic malformations, in experimental animals [3]. Although the exact mechanism is not yet fully elucidated, emerging evidence indicates that HU damages the reproductive system through two converging pathways [4]. Mechanistically, hydroxyurea quenches the tyrosyl radical of ribonucleotide reductase, depleting intracellular deoxyribonucleotide pools and triggering replication-stress–induced DNA damage, cell-cycle arrest, and p53-caspase-3-mediated apoptosis in granulosa cells and oocytes. In parallel, its carbamoyl-nitroso intermediate generates excessive reactive oxygen species that depolarise mitochondria, deplete glutathione, and exacerbate oxidative injury within the follicular micro-environment, further amplifying cell death [5].

Current treatments for DOR-related infertility caused by HU include pretreatment with drugs such as dehydroepiandrosterone and coenzyme Q10, controlled ovarian hyperstimulation, in vitro fertilization and embryo transfer, hormone replacement therapy, ovarian injections of platelet-rich plasma, but evidence for the efficacies of these approaches is currently lacking and patient conception rates are generally < 40% [6]. Furthermore, the past years have witnessed considerable advances in the knowledge base related to the use of stem cells for regenerative medicine. Recent breakthrough discoveries in stem cells and cell-free therapy have made them an ideal source for future cell therapy in DOR, premature ovarian failure (POF)/premature ovarian insufficiency (POI). Although animal testing and preclinical studies have shown the effectiveness of these therapeutics, their clinical applications still have limitations, including insufficient cell sources, unstandardized treatment protocols, ethical issues and side effects [7]. Compared with modern therapy, Traditional Chinese Medicine (TCM) has a history of thousands of years and has developed a comprehensive theoretical system and diagnostic and treatment methods. Chinese herb contains a variety of ingredients, they can minimize side effects while maintaining efficacy through multi-target and multi-pathways. TCM may provide an effective strategy to alleviate HU-induced DOR.

In the theory of TCM, gastrointestinal belongs to the category of “spleen” in traditional medicine. Thousands of years ago, ancient Chinese scholars summarized the relationship between spleen and reproductive functions. For example, “Yellow Empero’s Canon Internal Medicine”, a classic ancient book, explained that damaged spleen cannot convert nutrients into Qi (energy) and blood, causing amenorrhea or even infertility in women [8]. Recent evidence suggests that the intestinal flora may be an important environmental factor contributing to abnormal reproductive system function. Fecal sex hormone concentrations in germ-free (GF) mice were lower than in specific pathogen-free (SPF) mice, however, after colonizing GF mice with the microbiota of SPF mice, the fecal hormone levels of the GF mice increased [9]. Multiple mechanistic studies have identified a possible correlation with β-glucuronidase production by gut microbes [10]. In addition, the gut microbiome and its metabolites, such as short-chain fatty acids, have been implicated in inflammation and immunity, and may likewise play a key role in reproductive disorders [11]. Elgart et al. found that Drosophila gut bacteria could inhibit egg formation, possibly associated with deficiency of intestinal acetate [12].

Zi Chong granules was derived from classic TCM prescriptions Zuogui Pills and Yougui Pills, both of which were recorded in *Jing Yue Quan Shu* (A.D. 1624) and consists of *Rehmannia glutinosa (Gaertn.) DC.*,* Dioscorea oppositifolia L.*,* Cornus officinalis Siebold & Zucc.*,* Lycium chinense Mill.*,* Cuscuta chinensis Lam.*,* and Cervi cornu degelatinatum.* Zi Chong granules have been used in clinical practice for over 20 years and have shown efficacy in patients with DOR, mostly due to ovarian insufficiency caused by a decrease in the number or quality of oocytes, accompanied by reduced levels of sex hormones and AMH and a decrease in the number of sinus follicles (AFC). In the past ten years, our research group has continued to clarify the efficacy of Zi Chong (ZC) granules in ovarian reserve function through clinical observations. Some studies have included infertile patients with premature ovarian failure, premature ovarian insufficiency, and endometriosis follicular development disorders. The controlled pre-post study was conducted. Compared with pre-treatment, ZC granules promoted the growth and development of antral follicles and endometrial hyperplasia in patients. The improvement of ovarian dysfunction may be related to the improvement of blood supply to the ovaries and uterus [13]. In addition, for patients with premature ovarian insufficiency, ZC granules can increase AMH, reduce follicle stimulating hormone (FSH), and increase the number of antral follicles, suggesting a tendency to improve ovarian function [14]. The above studies all provide scientific basis for ZC granules to improve ovarian function. A previous study found that ZC granules could promote estrogen secretion from granulosa cells and follicle development in mice [15]. ZC granules also induced the differentiation of human embryonic stem cells to granulosa cells, and the promotion of granulosa cell differentiation and development by this compound reinforced its positive regulatory effect on follicular development [16]. In addition, intestinal infusion of ZC granules improved follicular development in young rats and increased estradiol (E_2_) levels in ovariectomized mice, showing estrogen-like effects [17]. These results suggest that ZC may improve reproductive function by regulating the intestinal microenvironment. However, it is not clear if ZC granules can mitigate HU-induced reproductive toxicity to restore fertility in DOR mice, and its potential mechanism of action via the intestinal bacteria also remains unclear.

This study therefore aimed to observe the effects of HU gavage on the reproductive system and investigate the pharmacodynamic effects of ZC granules on reproductive function in mice. We also used 16 s rDNA and targeted metabolomics techniques to detect the intestinal bacteria and blood metabolites in mice before and after HU gavage, and the differential intestinal bacteria and metabolic pathways regulated by ZC granules, in order to explore the mechanism of HU reproductive toxicity and the restoration of fertility by ZC granules following DOR.

## Methods

### Chemicals and reagents

The following chemicals and reagents were obtained from the noted sources: HU tablets (Qilu Pharmaceutical Co., Ltd., Shandong, China), pregnant mare serum gonadotrophin (PMSG; Solebo Technology Co., Ltd., Beijing, China; no.: 20190602), human chorionic gonadotropin (HCG; Lizhu Group Lizhu Pharmaceutical Factory, Zhuhai, China; no.: 11301010030 C), mouse E_2_ enzyme-linked immunosorbent assay (ELISA) kit (LMAI Bio, Shanghai, China; no.: LME2020021009), mouse AMH ELISA kit (LMAI Bio, Shanghai, China; no.: LME2020022010), mouse progesterone ELISA kit (LMAI Bio, Shanghai, China; no.: LME202002091), M2 culture medium(Nanjing Aibei Biotechnology Co.,Ltd, Nanjing, China; no.: 1912 A), Hyaluronidase from bovine testes(Sigma, US; no.: H3884), anti-CD34 antibody (Boster, Wuhan, China; no.: BA3414), anti-estrogen receptor α (ERα) antibody (Abcam, UK; no.: ab92516), anti-progesterone receptor α (PRα) antibody (Abcam, UK; no.: ab101688), anti-Syndecan-1 (CD138) antibody (Abcam, UK; no.: ab128936).

### Preparation of ZC granules

 As shown in Table 1, ZC granules comprises *Rehmannia glutinosa (Gaertn.) Libosch ex Fisch.et Mey.*,* Dioscorea opposita Thunb.*,* Cornus officinalis Sieb. et Zucc.*,* Lycium chinense Mill.*,* Cuscuta chinensis Lam.*,* Cervi cornu degelatinatum.* The plant name has been checked with Medicinal Plant Names Services (MPNS, http://mpns.kew.org). The herbs were mixed in a ratio of 3: 3: 3: 3: 3: 1 and treated with two rounds of extraction using boiling water. The extracts were then combined and filtered and concentrated under reduced pressure to produce a paste with a density of 1.30 g/cm^3^. The dregs were dried and finely powdered, and then mixed evenly with the paste. The mixture was sprayed into granules and dried. One dose yielded 8.4 g dry powder.

**Table 1:** The compositions of ZC granules

Chinese name	Accepted scientific name	Family	Plant part	Batch number	Amount(g)
Shudihuang	*Rehmannia glutinosa (Gaertn.) Libosch ex Fisch.et Mey.*	Orobanchaceae	Root	2,005,005 S	15 g
Shanyao	*Dioscorea opposita Thunb*	Dioscoreaceae	Root	2,006,003 C	15 g
Shanzhuyu	*Cornus officinalis Sieb. et Zucc.*	Cornaceae	Fruits	2,006,003 C	15 g
Gouqizi	*Lycium chinense Mill.*	Solanaceae	Fruits	2,004,006 S	15 g
Tusizi	*Cuscuta chinensis Lam.*	Convolvulaceae	Seed	2,007,001 S	15 g
Lujiaoshuang	*Cervi cornu degelatinatum*	Cervidae	Cornu cervi	2,003,001 S	5 g

### Chemical composition analysis of ZC granules

The chromatographic analysis of ZC granules was carried out according to the 2015 edition of the Chinese Pharmacopoeia, performing on SHIMADZU LC-40BX3 (SHIMADZU, Japan), using Ultimate UHPLC XB-C18 column (4.6 × 100 mm, 1.8 μm). The crushed material was obtained by grinding the Zi Chong granules, and after repeated mixing, 2 g of the material was taken. It was then added to 20 ml of 50% methanol, vortexed for 10 min, subjected to ultrasonic extraction for 30 min, centrifuged, and the supernatant was collected. The volume was adjusted to 20 ml, and the mixture was filtered for UHPLC analysis. The mobile phases were acetonitrile (A) and water (B). The gradient elution condition was as follows: 0–20 min, 5%−30% A; 20–30 min, 30%−100% A; 30–35 min 100% A; 35–37 min 5% A; 37–38 min 5% A; the detection wavelength was 254 nm, the flow rate was 0.5 ml/min, the column temperature was 35℃, the injection volume was 5 µl.

### Animals

Seventy-two specific pathogen-free female Kunming mice, age 4–6 weeks, were purchased from Dashuo Experimental Animal Center (Sichuan, China; certificate no.: SCXK [Sichuan] 2020-030). All mice were housed at room temperature (22 ± 2℃), 45%–55% humidity, and a 12 h light/day cycle, with *ad libitum* access to food and water. This study was approved by the Experimental Animal Ethics Committee of Chengdu University of Traditional Chinese Medicine (2021DL-002). All experiments were performed in accordance with relevant named guidelines and regulations, and the author complied with Animal Research: Reporting of In Vivo Experiments (ARRIVE) guidelines.

### Experimental design

The mice were divided randomly into three groups: control group (CON), model group (HU), and HU + ZC group. Mice in the HU and HU + ZC groups were gavaged with HU suspension at a dose of 400 mg/kg/day [18, 19], and mice in the CON group were gavaged with an equal amount of saline, for a total of 21 days. Mice in the HU + ZC group were then gavaged with 2.5 g/kg ZC granules daily, and mice in the HU and CON groups were gavaged with the same amount of saline, for a total of 15 days. To ensure uniformity among the groups, mice in the HU and HU + ZC groups were injected intraperitoneally, after gavage, with PMSG (5 IU) + HCG (5 IU) to promote ovulation, and vaginal smears were checked in the CON group to determine the estrous cycle. Mice were anaesthetized with pentobarbital sodium salt (3 mg/ml), at 0.01 ml/g mouse body weight, adjust with saline to 15 ml volume and administered intraperitoneally.

For the first procedure, 12 mice were selected randomly from each group. Mice in the HU and HU + ZC groups were sacrificed under anesthesia at 12 h and 72 h post-ovulation, and mice in the CON group were sacrificed before and after ovulation, respectively. Blood was collected and the ovaries and uterus were removed and fixed in 4% paraformaldehyde buffer for histopathological examination. The day before sacrifice, feces were collected from the mice using sterilized equipment and then frozen rapidly in liquid nitrogen for 16 S rDNA gene sequencing.

For the second procedure, six mice were selected randomly from each group. Post-ovulation mice in the HU and HU + ZC groups and CON mice in estrus stage were arranged in a 2:1 female: male ratio for mating.

For the third procedure, six mice were selected randomly from each group. Mice in the HU and HU + ZC groups were anesthetized and sacrificed 16 h after ovulation, and mice in the CON group were anesthetized and sacrificed after detection of a vaginal plug. The bilateral oviducts were removed and placed in 35 mm dishes to obtain oocytes for staging and counting.

### ELISA

Serum concentrations of AMH, E_2_, and progesterone were detected by ELISA, according to the instructions in the respective kits.

### Superovulation

After the modeling and drug intervention, mice in the HU and HU + ZC groups were treated with an intraperitoneal injection with 5 IU of PMSG at 16:00, and 48 h later with 5 IU of HCG intraperitoneally. On the following day at 8:00, the mice were anesthetized and sacrificed. The mice in CON group were anesthetized and sacrificed when they were in proestrus. This PMSG/hCG regimen not only yields mature oocytes but also serves as a reliable estrous-cycle synchronization protocol in chemically treated mice.

### Oocyte staging and counting

Bilateral oviducts from mice in each group were put into 35 mm dishes and gradually torn to release all the oocytes. The oocytes were put into a culture dish containing hyaluronidase to separate the granulosa cells, and the oocytes separated from the granulosa cells were then put into a culture dish containing M2 culture medium and staged and counted under the microscope. MII stage oocytes were identified by uniform cytoplasm, small perivitelline gaps, discharged first polar body and smooth surface, and a clear and uniform zona pellucida. Abnormal oocytes were characterized by an irregular shape, refractive areas or large vacuoles in the cytoplasm, and a thickened or raised zona pellucida.

### HE staining of ovary

Ovaries fixed in 4% paraformaldehyde were rinsed in running water for 30 min, trimmed and processed through graded ethanol (75% 6 h, 85% 10 h, 95% 4 h, absolute ethanol Ⅰ 2 h, absolute ethanol Ⅱ 2 h), cleared in xylene (Ⅰ 20 min, Ⅱ 15 min), infiltrated with paraffin for 3 h and embedded. Section (5 μm) were cut on a Leica RM2235 microtome, floated on warm water, mounted, and baked at 60 °C for ≥ 2 h. After de-paraffinisation, slides were stained with hematoxylin for 30 min, rinsed 20 min, differentiated with acid alcohol, counter-stained with eosin for 5 min, dehydrated in graded ethanol, cleared in xylene and sealed with neutral resin. Follicle and corpus luteum counts were obtained from 20 random fields using Image-Pro Plus 6.0.

### Microvessel density (MVD) immunostaining of uterus

 Paraffin sections were de-paraffinised and rehydrated, followed by microwave antigen retrieval in pH 6.0 citrate buffer (8 min × 2, high power). Endogenous peroxidase activity was blocked with 3% H₂O₂ for 15 min. After phosphate-buffered saline (PBS) washes, slides were incubated in a humid chamber with anti-CD34 antibody (1:200, Boster, BA3414) at 37 °C for 45 min, followed by the EnVision™ + HRP polymer (ChemMate, Agilent) for 45 min. Colour development used diaminobenzidine (DAB), nuclei were counter-stained with hematoxylin, and slides were dehydrated, cleared and mounted. MVD was quantified as the mean of five hotspots (× 400) per section.

### ER-α & CD138 IHC (uterus)

Section (4 μm) were prepared as above, de-paraffinised, and subjected to antigen retrieval in citrate buffer (pH 6.0). After blocking with normal goat serum for 15 min, slides were incubated overnight at 4 °C with anti-ER-α (1:300, Abcam ab92516) or anti-CD138 (1:250, Abcam ab128936). After PBS washes, biotinylated secondary antibody and HRP-streptavidin (ZSGB-BIO, SP-9001) were applied sequentially for 15 min each at room temperature. DAB was used for chromogenic detection, followed by hematoxylin counter-stain, dehydration and mounting. ER-α expression was semi-quantified using the H-score (intensity × percentage of positive cells). CD138 positivity was expressed as CD138% = positive (brown) nuclei/total nuclei × 100%. Images were acquired with an Olympus CX22 microscope and analysed in Olympus CellSense.

### Histopathological analysis of ovarian and uterus tissues

The tissues were fixed in 4% paraformaldehyde buffer, serially dehydrated, embedded in paraffin, sliced into 5-µm thick sections, and stained with hematoxylin-eosin (HE) and sealed with resin glue. The tissue samples were then photographed using a microscope imaging system, and primordial and atretic follicles were counted in the images using Image Pro Plus 6.0. Twenty different views were selected, and the endometrial thickness and glandular area were measured in each view and averaged.

In addition, uterine tissue sections were stained immunohistochemically to determine the MVD and CD138 and ER expression. Sections were repaired using pH6 citrate antigen repair solution, rinsed with PBS, incubated with peroxidase blocker (3% H_2_O_2_), rinsed again with PBS, and incubated with primary antibodies at 37℃, followed by rinsing with PBS, incubation with solution A from a two-step anti-rabbit/mouse universal immunohistochemical assay kit (ChemMate™ Envision + HRP) at 37℃, and rinsing with PBS. The sections were then incubated with chromogenic DAB working solution, re-stained with hematoxylin, dehydrated through graded alcohols, transparentized with xylene, and sealed with neutral glue. The positive judgement of sections was from the perspective of the staining intensity and the percentage of positive cells in sections.

### 16 s rDNA gene sequencing of feces

Total DNA was extracted from feces samples. Primers were designed according to the conserved region, and a sequencing junction was added at the end of the primers. Polymerase chain reaction amplification was then performed and the products were purified, quantified, and homogenized to form a sequencing library. The built library was subjected to quality control and then sequenced using an Illumina HiSeq 2500. The original image data were obtained and transformed into raw sequences (sequenced reads) by base calling. The observed operational taxonomic units (OTUs) were clustered according to 97% similarity sequences using USEARCH (version 11.0.667, http://www.drive5.com/usearch/) software. Sequencing data were interpreted using Quality Interactive Metadata Inference Environment (QIIME2, version 2021.2). Principal coordinate analysis (PCA) was performed using the R package to show the β-diversity of the microbiome between samples. Bacterial classifications were compared among the groups using Wilcoxon’s rank sum test. Based on the 16 S rDNA gene sequencing data, the relative abundance of microbial functional categories in the samples was predicted using the Phylogenetic Investigation of Communities by Reconstruction of Unobserved States (PICRUSt) software [20]. Subsequently, the significance of the PICRUSt2 predicted results was tested using the limma package in R software for differential analysis of Kyoto Encyclopedia of Genes and Genomes (KEGG) metabolic pathways (*p* < 0.05 was considered significant).

### Non-targeted metabolomics assays

A 100 µL serum sample was mixed with 3 vol of pre-cooled acetonitrile, shaken at room temperature for 10 min, chilled at −20 °C for 30 min, centrifuged at 14 000 g, 4 °C, 15 min, and the supernatant was transferred to an LC-MS vial.

### HPLC conditions (metabolomics)

Chromatographic separation was carried out on a Vanquish UHPLC system (Thermo Fisher) equipped with a Hypersil GOLD C18 column (100 mm × 2.1 mm, 1.9 μm; 40 °C; 0.20 mL min⁻¹; injection volume 2 µL). Mobile phases were 0.1% formic acid in water (A) and methanol (B) for positive-ion mode, and 5 mM ammonium acetate (pH 9.0) (A) and methanol (B) for negative-ion mode. The gradient was 0–1.5 min 98%A; 1.5–12 min linear to 0%A; 12–14 min 0%A; 14–17 min 98%A.

### Mass spectrometry

Detection was performed on a Q Exactive HF mass spectrometer (Thermo Fisher) operating in full MS/data-dependent MS² mode (m/z 100–1500) with ESI parameters: spray voltage 3.2 kV, capillary 320 °C, sheath gas 40, aux gas 10. Raw spectra from positive- and negative-ion modes were processed with Compound Discoverer (v 1.0). Metabolite annotation used the mHU + ZCloud and ChemSpider databases; additional matching against the mzVault OTCML high-resolution library was also performed.

### Statistical analysis

All data are presented as mean ± standard deviation (SD). Normality was assessed with the Shapiro–Wilk test. For variables showing a normal distribution, differences among the three groups were evaluated by one way ANOVA in SPSS 25.0, followed by pair wise Holm step down post hoc tests; for non-normal data, the Kruskal–Wallis H test with Holm correction was applied. To control the family wise error rate (FWER) in high dimensional comparisons—namely bacterial taxa, metabolites, KEGG pathways and the three pair wise analysis of similarities (ANOSIM) tests—all raw p-values were adjusted with the Holm–Bonferroni procedure in R (p. adjust, method = “holm”), and Holm adjusted *p* < 0.05 was considered significant. Bray–Curtis distance matrices were computed with the vegan package (v 2.5.3); PCA based on these distances were also performed in vegan and visualized with ggplot2 (v 2.2.1). Community differences were assessed by ANOSIM (vegan, 9 999 permutations) and reported with Holm adjusted p-values. Finally, Spearman correlations between gut microbial genera and fecal metabolites were calculated with the psych package, and Holm adjusted p-values were used to mask non-significant cells in heat map visualizations produced with heatmap package.

## Results

### Chemical compositions of Zi Chong granules

**Fig. 1:**
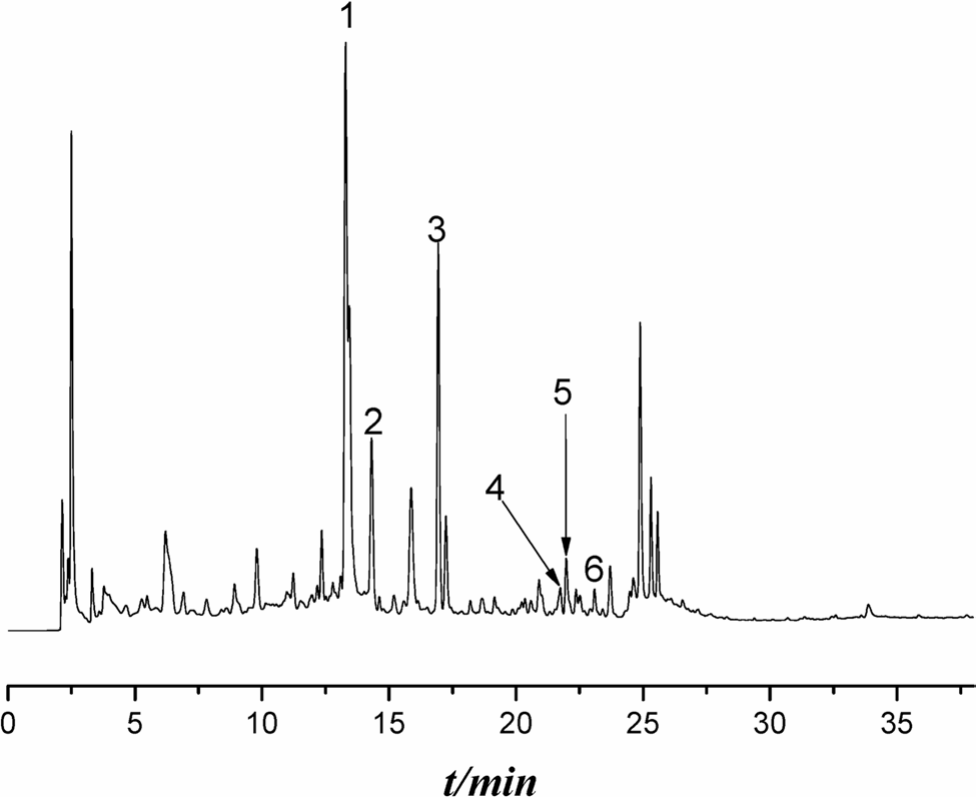
Chemical Compositions of Zi Chong granules. The HPLC chromatograms of ZC granules, including Morroniside (1), Chlorogenic acid (2), Loganin (3), Hyperoside (4), Isoquercitrin (5), Acteoside (6)

Phytochemical analysis of the ZC granules was conducted using an UHPLC (Supplementary Fig. 1). The chemical compound peaks of ZC granules were identified by standards based on the specific retention time of each compound. Six main compounds were identified (Fig. 1), including *Morroniside*,* Chlorogenic acid*,* Loganin*,* Hyperoside*,* Isoquercitrin*,* Acteoside.*

### ZC reversed ovarian reserve function in mice exposed to HU

**Fig. 2:**
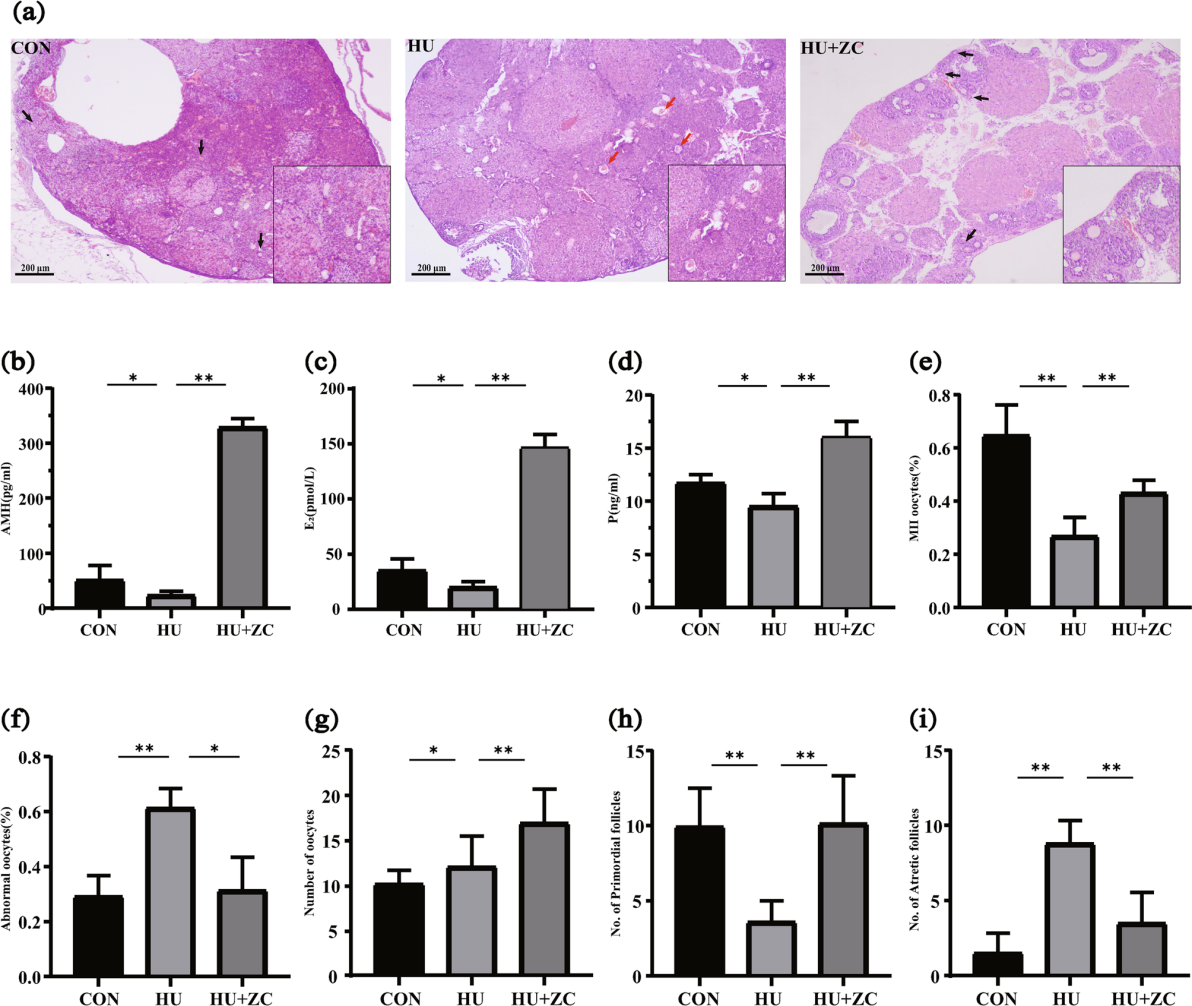
Effect of ZC granules on ovarian reserve function in HU-treated mice. **a** Ovaries stained with HE (× 50). Scale bar = 200 μm. Black arrows indicate primordial follicles; red arrows indicate atretic follicles. **b** AMH, (**c**) E_2_, and (**d**) progesterone levels. Numbers of (**e**) MII stage, (**f**) abnormal, and (**g**) total oocytes, (**h**) Number of primordial follicles, (**i**) Number of atretic follicles. *p < 0.05, **p < 0.01

We investigated the effect of HU on ovarian reserve function in mice and the ability of ZC granules to reverse the effect. Oocytes in the CON and HU + ZC groups mostly comprised a single layer of flattened follicular cells in the periphery, i.e., primordial follicles, while oocytes in the HU group mostly had indistinct or absent structures and the zona pellucida was crinkled and depressed, suggesting that they were mostly atretic follicles. These observations suggested that HU accelerated abnormal follicular apoptosis in mice, while ZC granules prevented excessive follicular atresia and hindered the depletion of primordial follicles by HU (Fig. 2a). The low AMH levels in the HU group compared with the CON group further indicated that HU reduced ovarian reserve, impaired ovarian function, and reduced oocyte quality. Compared with the HU group, AMH levels were increased in the HU + ZC group (Fig. 2b), indicating that ZC granules restored ovarian reserve function. We also examined estrogen and progesterone levels to assess ovarian function. Serum E_2_ levels were 34.66 ± 10.48 pmol/L in the CON group, but were decreased by about 37.19% in the HU group compared with the CON group, and increased about 6.71-fold in the HU + ZC group compared with the HU group. Progesterone levels showed a similar trend (Fig. 2c and d). In addition, staging and counting of expelled oocytes after COH showed significant decreases in total oocytes (*p* < 0.05) and stage MII oocytes (*p* < 0.01) and a significant increase in abnormal oocytes (*p* < 0.05) in the HU group compared with the CON group, all of which effects were reversed in mice treated with ZC granules (*p* < 0.01, *p* < 0.05, *p* < 0.01, respectively) (Fig. 2e-g). Compared to the Con mice, the HU mice showed lower numbers of primordial follicles and higher numbers of atretic follicles, a result that was reversed by ZC granules (*p* < 0.01, *p* < 0.01) (Fig. 2h-i).

### ZC improved endometrial receptivity in mice exposed to HU

**Fig. 3:**
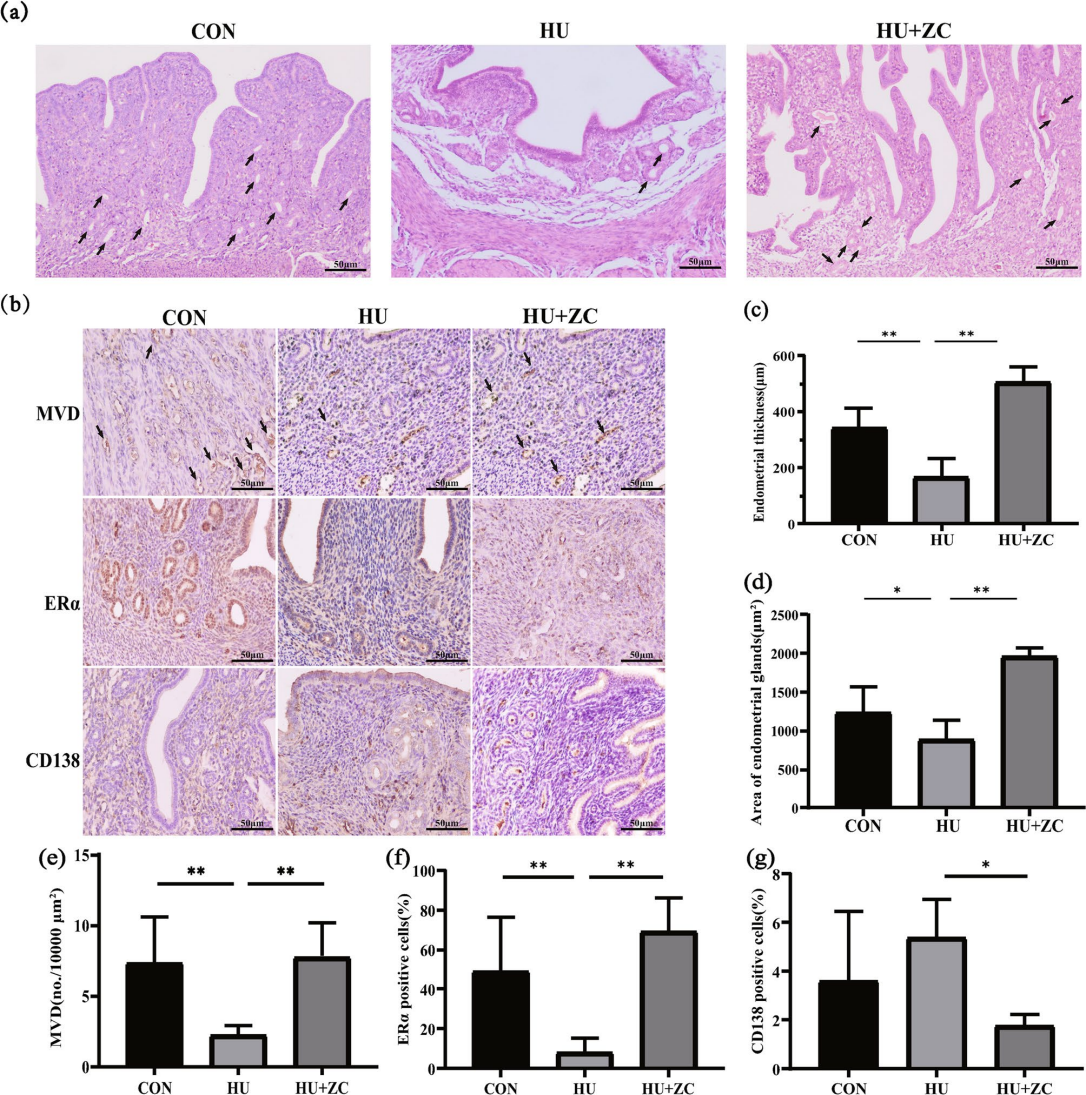
Effect of ZC granules on endometrial receptivity in mice exposed to HU. **a** Endometrial HE staining (× 200). Scale bar = 50 μm. **b** MVD, ERα, and CD138 immunohistochemical staining (× 200). Scale bar = 50 μm. (**c**) Endometrial thickness and (**d**) endometrial gland area. Corresponding quantitative analysis of (**e**) MVD, (**f**) ERα positive cells, and (**g**) CD138 positive cells. *p < 0.05, **p < 0.01

The endometrium is regulated by ovarian hormone levels, and low ovarian reserve function can lead to poor endometrial tolerance. We therefore examined the endometrium by HE staining. Compared with the HU group, the endometrium was thicker in the CON group (343.00 ± 70.09 μm), with more glands and dilated ducts and visible protein secretion, suggesting that HU resulted in a thinner endometrium. Compared with the HU group, mice in the HU + ZC group had a thicker endometrium with more glands but no significant dilation (Fig. 3a), suggesting that ZC granules could partly restore endometrial thickness. The endometrial thickness and glandular areas were quantified, and the results were consistent with the trends indicated in the images, with a significant difference between the groups (*p* < 0.01) (Fig. 3c and d). We further assessed the endometrial blood supply by determining the MVD, manifested as brownish-yellow granules in the cytoplasm of vascular endothelial cells. The granules were regular in morphology and uniformly distributed in the CON group, but were concentrated and decreased in density in the HU group. However, the granules showed normal morphology and increased density in the HU + ZC group compared with the HU group. Endothelial ERα levels detected by immunohistochemistry showed similar trends to MVD. We also carried out CD138 staining to assess the inflammatory status of the endometrium. CD138 was localized in the cytoplasm and cell membrane, with low expression in the endometrial mesenchyme in the CON group suggesting few plasma cells, compared with high expression in the HU group, suggesting more plasma cells, with reduced expression in the HU + ZC group compared with the HU group.

### Zi Chong granules regulated the dysbiosis of gut Microbiome species caused by hydroxyurea

**Fig. 4:**
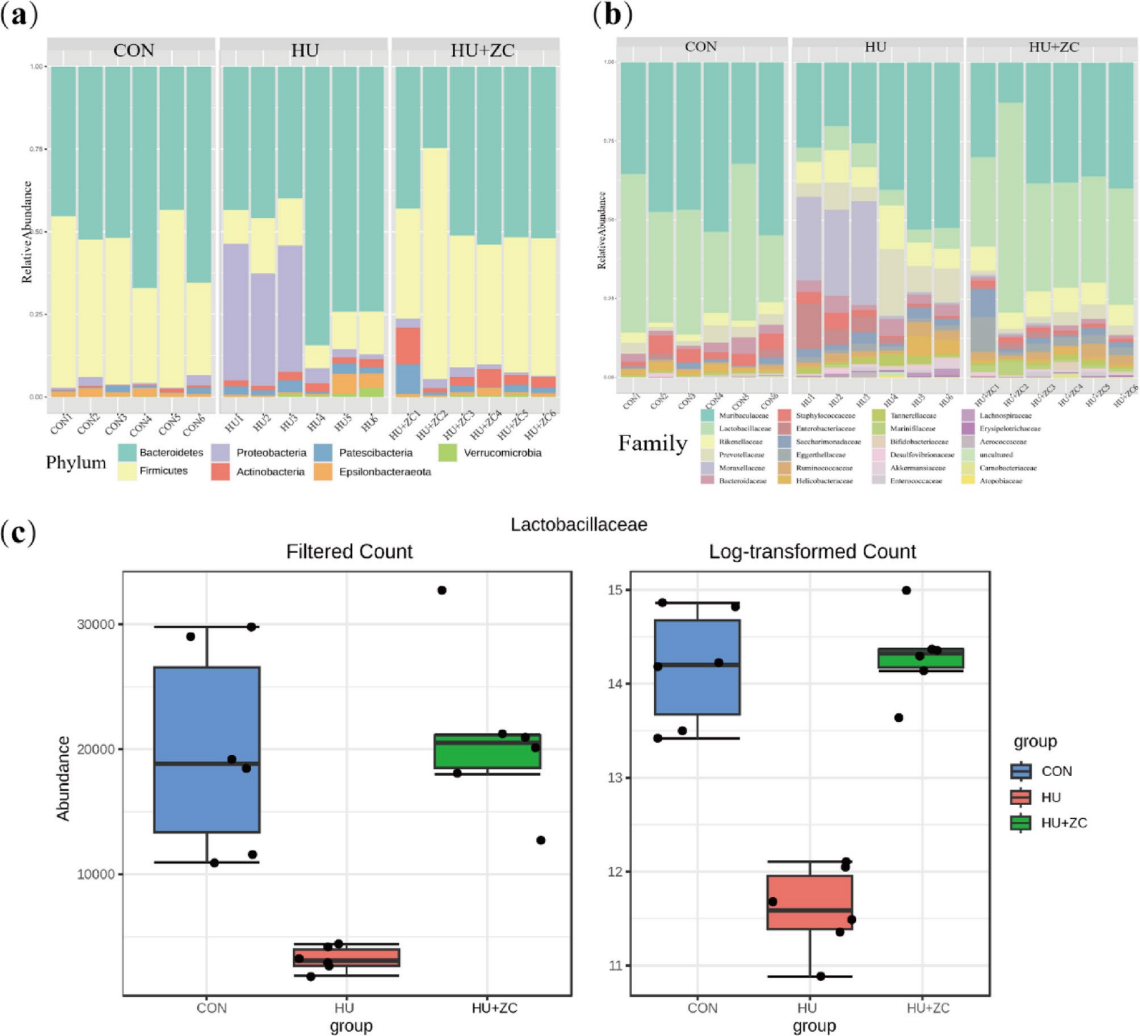
Composition of gut microbiota in mice treated with HU, HU + ZC, and CON, respectively, at the (**a**) phylum level and (**b**) family level. **c** Abundance (filtered count and log-transformed count) of Lactobacillaceae in mice treated with HU, HU + ZC, and CON, respectively

We investigated if the contribution of HU to the symptoms of DOR in mice and the improvement in symptoms following ZC treatment were related to the gut microbiota. We performed 16 S rDNA sequencing of fecal samples from mice in the HU, HU + ZC, and CON groups to characterize the differences in their gut microbiomes. Species stacking plots for the gut microbiome at the phylum level (Fig. 4a) showed that *Bacteroidetes* was the most common phylum in all groups. HU significantly decreased the proportion of thick-walled phyla compared with the CON group, and this change was reversed in the HU + ZC group. In addition, the proportion of *Proteobacteria* increased in the HU group relative to the CON group. At the family level (Fig. 4b), the proportion of *Lactobacillaceae* was significantly decreased while *Prevotellaceae* was significantly increased in the HU group compared with the CON group, with no significant differences in proportions between the HU + ZC and CON groups. The most significant changes were in the abundance of *Lactobacillaceae*, and both filtered and log-transformed counts showed that ZC granules improved the HU-impaired levels of intestinal microorganisms in mice (Fig. 4c). Overall, these results suggest that the addition of HU altered the composition of the gut microbiome in mice and that ZC granules reversed this effect.

### Zi Chong granules restored the structure of gut Microbiome in DOR mice

**Fig. 5:**
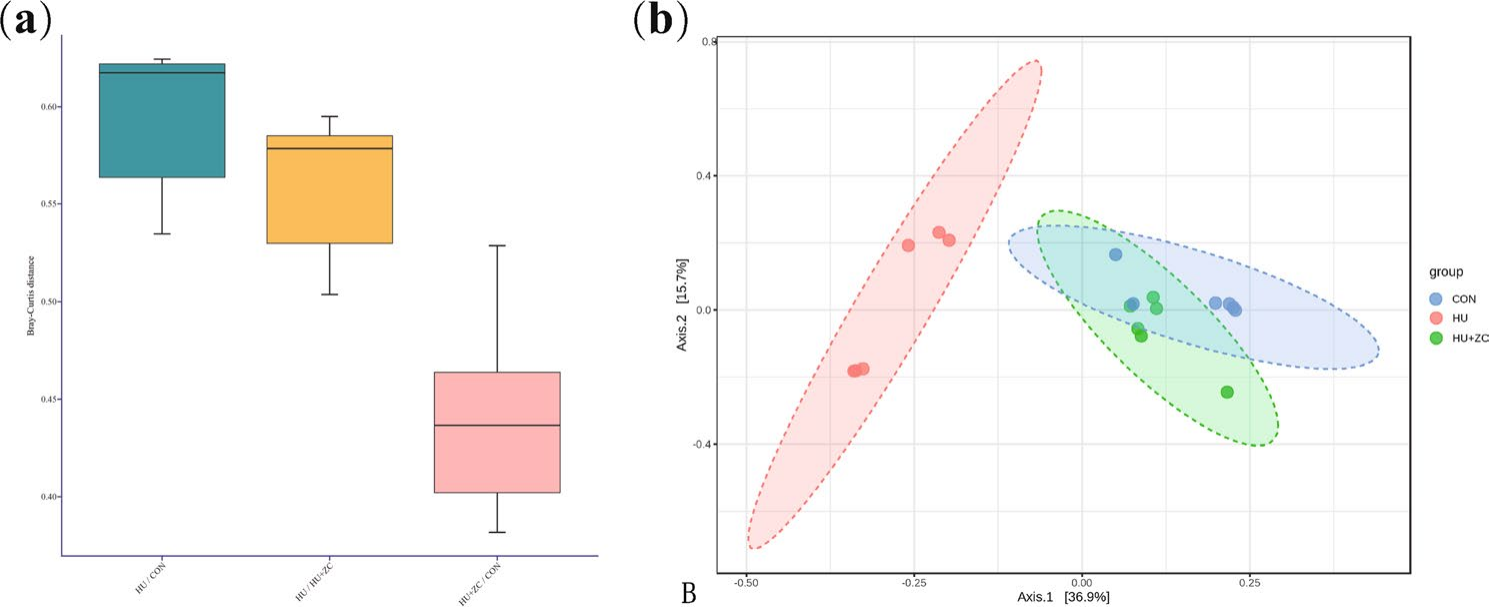
(**a**) Box plot of Bray–Curtis distance and (**b**) PCA plot of β-diversity of gut microbiome in mice treated with HU, HU + ZC, and CON, respectively

β-Diversity analysis of the gut microbiota based on OTU abundance was used to visualize the clustering of the microbial communities in each group. The box line plot of β-diversity (Fig. 5a) showed that the HU + ZC group was more similar to the CON group than the HU group was to the CON group. The PCA plot (Fig. 5b) showed that axis 1 (PCA1) explained 39.9% of the variability and axis 2 (PCA2) explained 17.2% of the variability. The PCA results showed that the gut microbial species differed significantly between the HU group and the CON group (*r* = 0.73, *p* < 0.01), with almost complete separation between the samples. The ANOSIM test results (Table 2) showed that the between-group differences were greater than the within-group differences, but the HU + ZC and CON groups had smaller R-values than the remaining two comparisons and showed a tighter aggregation.

**Table 2:** ANOSIM results for β-diversity of bacterial communities in mice treated with HU, HU + ZC, and CON, respectively

	HU/CON	HU/HU + ZC	HU + ZC/CON
R-value	0.944	0.798	0.566
P-value	0.003	0.002	0.003
Holm-adjusted *p*	0.006	0.006	0.006

### ZC granules restored the metabolome in DOR mice

**Fig. 6:**
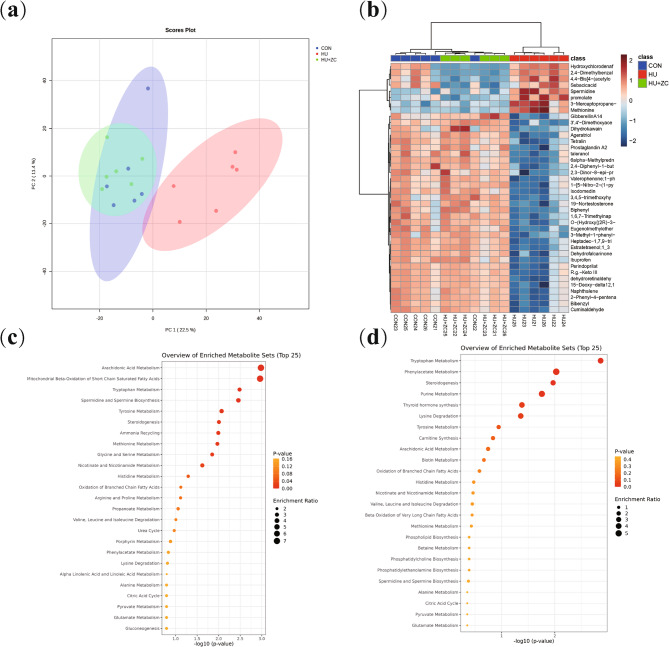
(**a**) Metabolome PCA analysis in mice treated with HU, HU + ZC, and CON, respectively. **b** Differential metabolite cluster heatmap. **c** Differential metabolite enrichment analysis between HU and CON groups and (**d**) differential metabolite enrichment analysis between HU + ZC and CON groups

We determined the effect of ZC granules at the metabolite level by examining the serum metabolomes in each group of mice by PCA analysis (Fig. 6a). The metabolome in the HU group was significantly separate from that in the CON group, while the metabolome in the HU + ZC group showed significant aggregation with the CON group (Table 3). This indicated that the CON group was more similar to the HU + ZC group at the metabolite level, suggesting that HU significantly altered the serum metabolome and that ZC granules had a significant restorative effect on the serum metabolome. A heatmap (Fig. 6b) showed that HU significantly decreased the levels of 40 metabolites and increased the concentrations of about 10 metabolites, while treatment with ZC granules significantly restored the levels of these metabolites, these metabolites can also be viewed in volcano plots (Supplementary Fig. 2a, b). KEGG enrichment analysis confirmed that HU gavage significantly altered many metabolic pathways (Fig. 5c), and that ZC granules restored these pathways, similar to levels in the CON group (Fig. 5d).

**Table 3:** ANOSIM results for PCA analysis of metabolome in mice treated with HU, HU + ZC, and CON, respectively

	HU/CON	HU/HU + ZC	HU + ZC/CON
R-value	0.537	0.687	0.13
P-value	0.004	0.004	0.016
Holm-adjusted *p*	0.012	0.012	0.016

### Correlation between gut microbiomes and follicle counts

**Fig. 7:**
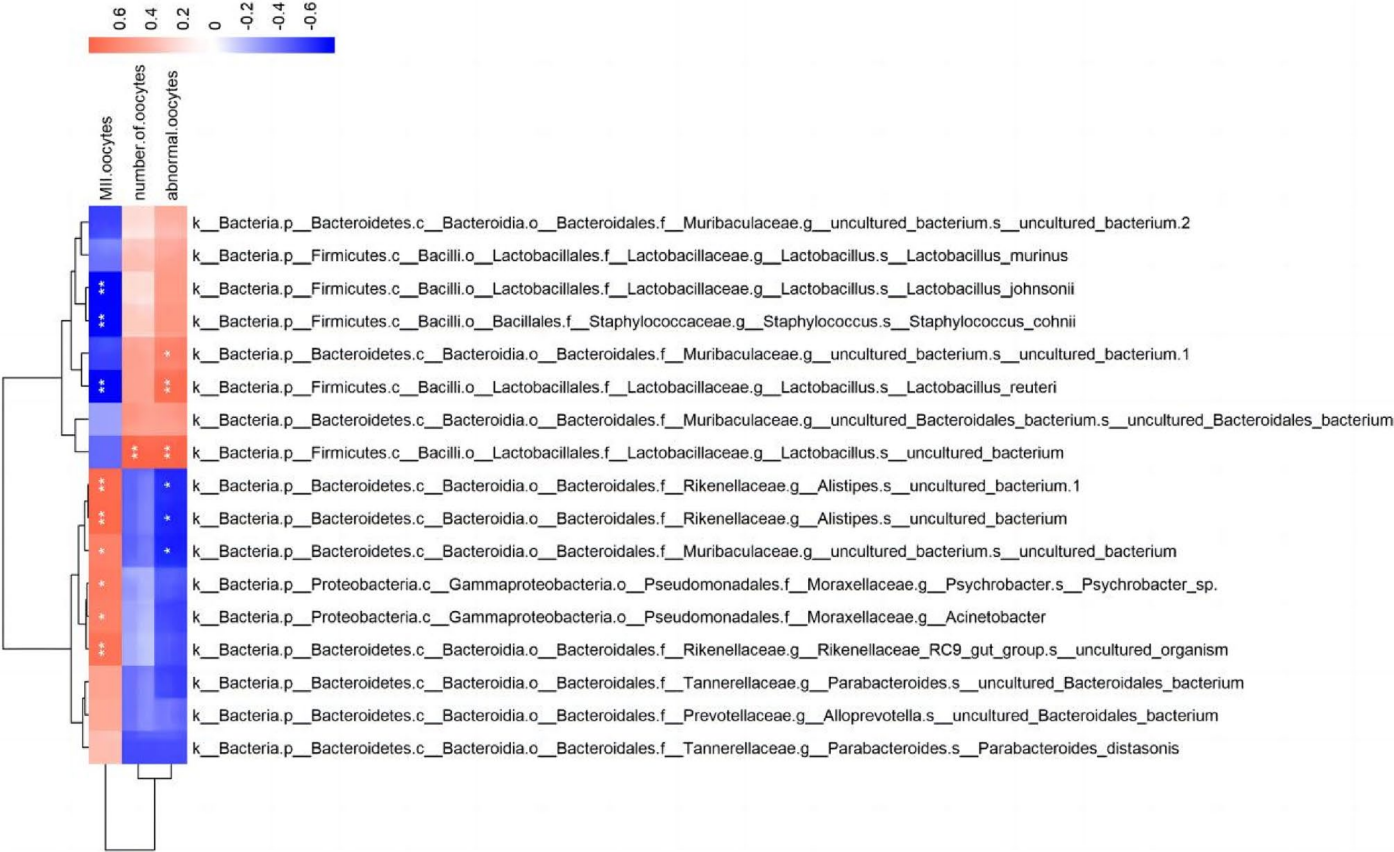
Correlation heat map between gut microbes and oocytes, calculated by Pearson correlation.The color gradient from red to blue represents the correlation between the microorganism and metabolite shifting from positive to negative, with darker colors indicating stronger correlations. *p < 0.05, **p < 0.01

To determine if HU-induced changes in gut microbes were responsible for DOR in mice, we examined the correlations of the different strains with the total numbers of oocytes, MII oocytes, and abnormal oocytes in the ovaries using Pearson’s correlation coefficient, and found that most of the significantly changed gut microbes were significantly correlated with the numbers of MII and abnormal oocytes (Fig. 7).

### Correlation between gut microbiomes and metabolome

**Fig. 8:**
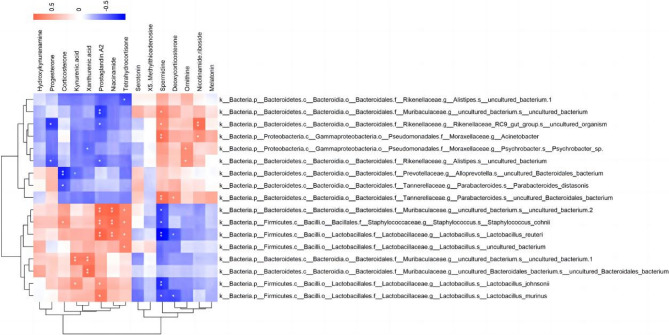
Heat map of correlation between gut microbes and metabolites, calculated by Pearson correlation

In order to determine which gut microorganisms were altered by HU and may thus affect metabolite levels, we examined the correlations between different microorganisms and serum metabolites using Pearson’s correlation coefficients, to further investigate the specific mechanism by which HU decreased ovarian reserve function. Notably, *Lactobacillaceae* showed a strong positive correlation with most metabolites (Fig. 8).

## Discussion

The results of this study demonstrated significant differences between HU-treated and untreated mice, with HU reducing ovarian reserve function, lowering sex hormone levels, and inducing endometrial hypotolerance. We further investigated the mechanism responsible for the HU-induced decrease in ovarian reserve function in mice by analyzing the gut microbiome and serum metabolome. ZC granules effectively improved the HU-induced low ovarian reserve function in mice, and this improvement was closely related to alterations in metabolic pathways caused by improvements in intestinal microbiology.

DOR is caused by a decrease in the number and/or quality of oocytes, leading to inadequate ovarian function and resulting in reduced fertility, accompanied by decreased AMH levels and a decreased AFC [21]. In this study, HE staining showed a decrease in the number of primordial follicles and increase in the number of atretic follicles in the ovary in HU-treated mice, as well as decreases in mature oocytes obtained by COH and an increase in abnormal oocytes, indicating a decrease in the number and quality of oocytes in mice. E_2_ is a steroid that is produced from androstenedione in the follicular membrane in follicular cells through the metabolism of ovarian granulosa cells hormones [19], while progesterone is an endogenous steroid secreted by ovarian luteal cells formed by follicles that have expelled oocytes after ovulation in the female ovary. The low serum E_2_ and progesterone levels in HU-treated mice suggest that HU induced ovarian functional impairment in DOR mice. Overall, these results suggest that HU caused reproductive toxicity and DOR in mice. AMH is a member of the transforming growth factor-β family. It is expressed in follicular granulosa cells in the ovaries of reproductive-age women and plays an important role in follicular growth and development by controlling the formation of primary follicles, via inhibiting the recruitment of excess follicles by follicle-stimulating hormone [21]. Changes in AMH precede changes in follicle-stimulating hormone and E_2_, and changes in AFC can reflect the numbers of sinus and antral follicles in the ovary and indicate ovarian reserve function [22, 23]. The decrease in serum AMH levels in mice after HU intervention corroborates the decline in ovarian reserve function induced by HU in mice.

The uterus is a direct target organ for estrogen and progesterone. Estrogen and its receptor bind to the endometrium and subsequently activate various protein factors in the nucleus to initiate mRNA transcription, activate cell mitosis, and regulate endometrial thickness and function. The ER is the main driver of estrogen action, and ER-deficient mice were shown to have a dysplastic and infertile uterus [24]. Mice with epithelial-specific deletion of ER-α exhibited abnormal expression of estrogen-responsive genes and failure of implantation [25]. A prospective clinical study also found that women with low endometrial thickness exhibited abnormal ER expression patterns and differential expression of genes that bind to the ER, and indicated that these genes may play a role in implantation by affecting proliferation and angiogenesis [26]. MVD reflects the number of microvessels per unit volume and is determined by measuring specific antigens, such as the endothelial cell adhesion factor CD34, to count blood vessels and determine the MVD as a quantitative measure of angiogenesis [27]. The MVD in a tissue reflects the abundance of the blood supply to the site. CD138 is a recombinant protein, a plasma cell-specific indicator, and a transmembrane proteoglycan expressed in stratified epithelium versus simple epithelium, and elevated levels of CD138 reflect inflammatory changes in the endometrium that are detrimental to embryo implantation [28]. In the present study, endometrial thickness was reduced and the glandular area was decreased in HU-treated mice, suggesting endometrial insufficiency, possibly associated with reduced estrogen and progesterone levels due to low ovarian reserve function, and further reducing endometrial ER expression and inhibition of endometrial vascular growth, resulting in an inadequate endometrial blood supply and an inflammatory state.

The administration of ZC granules effectively increased the number of oocytes, improved oocyte quality, increased AMH levels, reversed the declining ovarian reserve function, increased E_2_ and progesterone levels, and restored the reproductive and hormone-secreting potential of the ovaries. The elevated hormone levels led to elevated expression of ERs in the endometrium, an enhanced blood supply, suppression of the HU-induced inflammatory state of the endometrium, and restoration of endometrial function, thus eventually restoring the reproductive ability of the mice. Nevertheless, these results do not rule out the possibility that ZC granules also exert direct ovarian effects that operate independently of the gut–ovary axis.

HU-induced DOR has been reported, but its effects on the gut microbiota and serum metabolome remain unclear. Drug intake is an important factor affecting the gut microbiota and metabolism, and HU, as a potent chemotherapeutic agent, may have important effects on these factors. We investigated this hypothesis by 16 s sequencing of fecal samples from mice 21 days after drug administration and by analysis of the serum metabolome. Analysis of β-diversity based on Bray–Curtis distances showed that HU significantly affected the gut microorganisms in mice, as revealed by the significant separation of the HU and CON group samples in PCA. The results for the serum metabolome showed similar significant differences to the gut microbiome, indicating that HU significantly affected both the gut microbiome and the serum metabolome. Correlation analysis also revealed a significant correlation between the gut microbiome and the follicle count in mice, suggesting that changes in the gut microbiome and metabolome might contribute to the decrease in ovarian reserve function. ZC granules improved the HU-induced effects on the gut microbiota and metabolites, restoring their β-diversity close to the control group; the composition of the gut microflora and the corresponding metabolite levels following treatment with HU and ZC granules were not significantly different from the normal state, and were significantly different from those in HU-treated mice without ZC granules. The significant effects of HU and ZC granules suggest that the decrease in ovarian reserve function caused by HU exposure, and the amelioration of this condition by ZC granules, can be explained by changes in intestinal microbes and metabolites affecting ovarian pathology.

We further analyzed the intestinal flora and metabolic differences in mice treated with HU. A species stacking plot showed that the proportion of *Lactobacillaceae* was most-significantly reduced in the HU group compared with the CON group. *Lactobacillaceae* are important probiotics in the intestinal tract and are relevant for intestinal ecological stability and health. *Lactobacillus spp.* have been reported to produce lactic acid, bacteriocins, and hydrogen peroxide in the endometrial microbial environment, to inhibit pathogens and establish a favorable environment for embryo implantation; however, the effect of *Lactobacillus spp.* in the intestinal flora on reproduction has not been studied [29, 30]. We identified 897 metabolites in metabolome samples and analyzed the differences between the HU and CON groups by univariate analysis. We found that prostaglandin A2 (PGA2), nicotinamide riboside, niacinamide, serotonin, kynurenic acid xanthurenic acid, melatonin, hydroxykynurenamine, deoxycorticosterone, tetrahydrocortisone, corticosterone, progesterone, ornithine, 5’-methylthioadenosine, spermidine, and 15 other metabolites were significantly differentially expressed between the CON and HU groups. Enrichment analysis of these differential metabolites screened by *t-*tests identified several differential metabolic pathways, including arachidonic acid metabolism, tryptophan metabolism, spermidine and spermine biosynthesis, steroidogenesis, and nicotinate and nicotinamide metabolism, as associated with decreased ovarian reserve function.

PGA2 plays a key role in arachidonic acid metabolism, via its G protein-coupled cell surface receptor, to affect oocyte maturation, ovulation, and volume expansion. PGA2 levels were significantly lower in the HU group compared with the CON group in the current study. Low levels of PGA2 inhibit the maturation of oocytes in the ovary, preventing secondary follicles from developing into mature follicles, thus leading to a decrease in the number of recruitable follicles in the ovarian cortical area and a decrease in oocyte quality, and thus to decreased ovarian reserve function [31].

Tryptophan metabolism may affect oocyte development and follicle quality through immunity. Tryptophan can be converted to melatonin, which can in turn delay the aging of oocytes in post-ovulatory mice via silent information regulator 1 (SIRT1) mitochondrial manganese superoxide dismutase (MnSOD)-dependent pathway, and can improve the inhibitory effect of bisphenol A on oocyte meiosis and fertilization and improve oocyte quality. HU downregulated the tryptophan metabolic pathway and reduced pathway activity, thus exerting a similar effect to downregulation of the arachidonic acid metabolism pathway [32].

Putrescine in the spermidine and spermine metabolic pathway is a precursor of spermine synthesis and has been reported to play an important role in granulosa cell luteinization. High levels of putrescine are also produced during ovulation in the ovary. HU decreased putrescine levels in this metabolic pathway and increased levels of spermine, leading to abnormal ovulation. In addition, the few mature follicles that are generated cannot be expelled, thus blocking ovulation and exacerbating the consequences of inadequate ovarian reserve function [33].

Steroid hormones are involved in many biological and physiological functions. Cholesterol is a precursor of steroid hormone synthesis. The levels of steroid hormones affect follicular growth and development. Wang et al. carried out bioinformatics analysis and showed that steroid-related genes were enriched in patients with reduced ovarian reserve function, suggesting that the steroid pathway may be related to this reduction in ovarian reserve. In the current study, we found that the steroid pathway was significantly affected in HU-treated mice, thus confirming that downregulation of this pathway reflected a decrease in ovarian reserve function. The downregulation of progesterone levels in the steroid pathway also indicated the downregulation of fertility, thus corroborating the decrease in ovarian reserve function [34].

Niacin and nicotinamide are two forms of water-soluble vitamin B3, also known as vitamin PP. As essential components of coenzymes, they are involved in anabolism and catabolism and play important roles in carbohydrate, lipid, and protein metabolism, and in the regulation of oxidative stress. Previous studies also indicated that niacin and nicotinamide metabolic pathways may be associated with decreased ovarian reserve function [29].

The above five metabolic pathways were either experimentally determined or bioinformatically predicted to be related to ovarian reserve function. The present results further corroborated the roles of these pathways as markers of ovarian reserve function by comparing their expression between DOR and normal mice.

The coincidental deviations of the gut microbiome and metabolome during treatment with HU and ZC granules suggest that changes in the metabolome might be correlated with changes in gut microorganisms. We therefore performed a correlation analysis to identify significantly changed gut microorganisms and metabolites, and found that all metabolites, except serotonin, were significantly correlated with gut microbes, strongly suggesting that the HU-induced decrease in ovarian reserve function was mediated via changes in metabolic pathways caused by altered gut microbes. The correlation between *Lactobacillus spp.* and PGA2 was of particular interest, due to their significant HU-induced changes in the intestinal microbial community and metabolite pathways, respectively, and their strong (*p* < 0.01) positive correlation, as reported previously [35, 36]. Prostaglandins (PGs) are generated by arachidonic acid (AA) via the cyclooxygenase (COX) pathway. AA is first catalyzed by COX to produce PGG_2_, which is subsequently metabolized to PGE_2_. PGE_2_ can then be dehydrated to generate PGA_2_ [20]. Many studies have reported that several species within the *Lactobacillaceae* family increase the release of COX-2 mediated PGE_2_ [37–40]. We therefore propose that HU gavage decreased the abundance of *Lactobacillus spp.* in the mouse intestine, which in turn resulted in downregulation of PGA2 levels. Low levels of PGA2 may then decrease arachidonic acid metabolism, resulting in inhibition of oocyte maturation in the ovary and a lack of progression to secondary follicles, ultimately resulting in a decrease in ovarian reserve function. In contrast, ZC granules can restore the abundance of *Lactobacillus spp.* and thus upregulate the activity of arachidonic acid metabolism, thereby restoring ovarian function.

There are some limitations in this study: First, the correlation analysis does not imply causation; therefore, antibiotic treatment or fecal microbiota transplantation experiments are still required to further validate our hypothesis. Second, while our study primarily focused on elucidating the mechanism of ZC granules through the gut-ovary axis, the gut-brain-ovary axis has attracted increasing attention in infertility research in recent years, which is suggested to be further explored in our future studies. Third, our work did not include clinical samples to validate the gut microbiota and metabolites, which is an important issue to be addressed in our next study to facilitate the translation from animal models to clinical applications.

## Conclusions

This study provides novel evidence for the effects of HU on the gut microbiome and metabolome, demonstrating a correlation between changes in the gut microbiome and changes in metabolic pathways. In addition, we demonstrated that the HU-induced decrease in ovarian reserve function was likely to be due to changes in metabolic pathways caused by the gut microbiome. The decrease in ovarian reserve caused by HU could be successfully reversed by ZC granules. The results of this study will help to clarify the pathogenesis of reduced ovarian reserve function and to target clinical treatment.

## Supplementary Information

Supplementary Material 1.

## Data Availability

The Illumina sequencing reads were uploaded to the SRA, https://www.ncbi.nlm.nih.gov/bioproject/PRJNA1070711/. Any other data supporting this study’s conclusions are available from the corresponding author on reasonable request.

## Abbreviations

AFC: Antral Follicle Counting
AMH: Anti-Müllerian Hormone
HU: Hydroxyurea
COH: Controlled ovarian hyperstimulate
CON: Control group
DOR: Diminished Ovarian Reserve
ER: Estrogen Receptor
E2: Estradiol
GF: Germ free
HE: Hematoxylin-Eosin
KEGG: Kyoto Encyclopedia of Genes and Genomes
MVD: Microvessel density
OTUs: Operational Taxonomic Units
PCA: Principal Coordinate Analysis
PGA2: Prostaglandin A2
SPF: Specific Pathogen-free
TCM: Traditional Chinese Medicine
ZC: Zi Chong granules
