# Supplementary material for: Therapeutic mechanisms of Zi Chong granules against hydroxyurea-induced diminished ovarian reserve based on integrated multi-omics analyses
Source: J Ovarian Res. 2025 Dec 16;18:295. doi: 10.1186/s13048-025-01846-5 (PMC12709716; doi:10.1186/s13048-025-01846-5)
Supplement: Supplementary file 1 — Supplementary Material 1. [file 13048_2025_1846_MOESM1_ESM.docx]

Supplementary Material

**Therapeutic mechanisms of Zi Chong granules against hydroxyurea-induced diminished ovarian reserve based on integrated multi-omics analyses**

Wenran Dong^1,8†^, Xinyu Guo^2†^, Hua Lu^1*^, Zhibin Liu^2^, Lan Xie^3,7^, Yi Liu^1^, Qian Wan^4^, Ren Chen^5^, Sui Liu^6^

***Correspondence：**Hua Lu: [sccd3@proton.me](mailto:sccd3@proton.me)


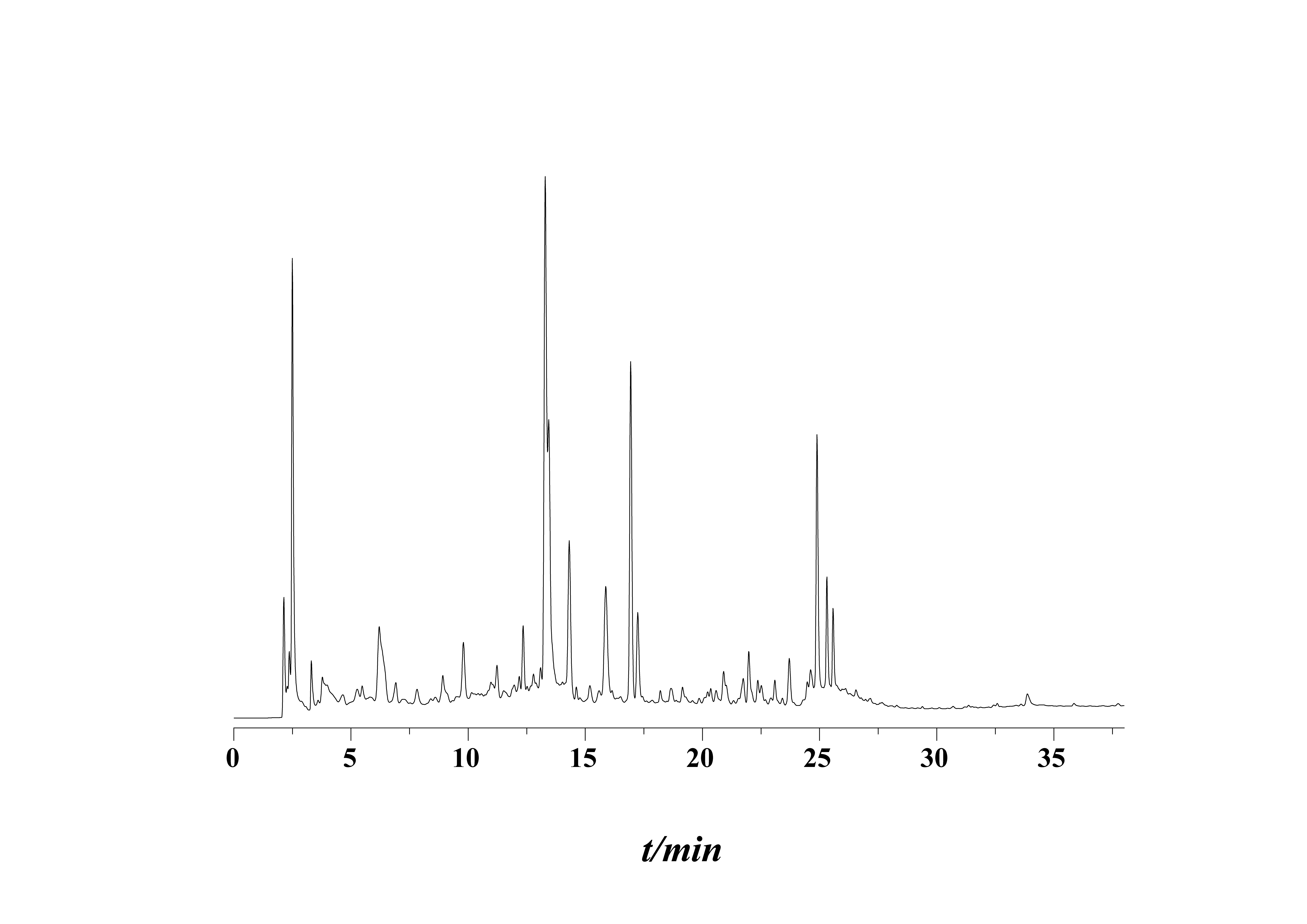


**Supplementary Figure 1.** UHPLC chromatograms of ZC granules.

**
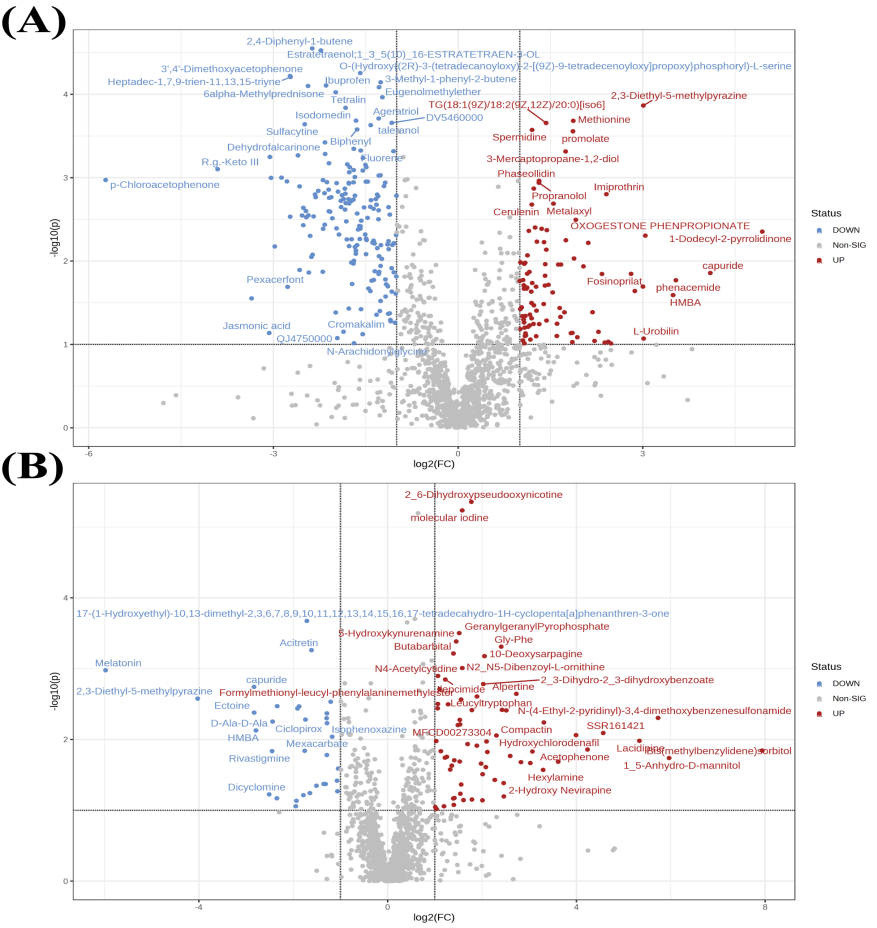
**

**Supplementary Figure 2.** (A) Volcano plot of different metabolites in HU group compared with CON group；(B)Volcano plot of different metabolites in HU group compared with HU+ZC group
